# Circulating 27-hydroxycholesterol, lipids, and steroid hormones in breast cancer risk: a nested case–control study of the Multiethnic Cohort Study

**DOI:** 10.1186/s13058-023-01693-6

**Published:** 2023-08-14

**Authors:** Mindy C. DeRouen, Juan Yang, Yuqing Li, Adrian A. Franke, Anne N. Tome, Kami K. White, Brenda Y. Hernandez, Yurii Shvetsov, Veronica Setiawan, Anna H. Wu, Lynne R. Wilkens, Loïc Le Marchand, Lenora W. M. Loo, Iona Cheng

**Affiliations:** 1 Department of Epidemiology and Biostatistics, School of Medicine, University of California San Francisco, 550 16th Street, San Francisco, CA 94538 USA; 2 Helen Diller Family Comprehensive Cancer Center, University of California San Francisco, 550 16th Street, San Francisco, CA 94538 USA; 3 Population Sciences of the Pacific Program, University of Hawaiʻi Cancer Center, Honolulu, HI USA; 4 Department of Population and Public Health Sciences, Keck School of Medicine, University of Southern California, Los Angeles, CA USA; 5 Norris Comprehensive Cancer Center, Keck School of Medicine, University of Southern California, Los Angeles, CA USA; 6 Cancer Biology Program, University of Hawaiʻi Cancer Center, Honolulu, HI USA; 7 Department of Preventive Medicine, University of Southern California, Los Angeles, CA USA

**Keywords:** 27-Hydroxycholesterol, Lipids, Steroid hormones, Breast cancer risk, Race and ethnicity

## Abstract

**Background:**

Laboratory studies have indicated that a cholesterol metabolite and selective estrogen receptor modulator, 27-hydroxycholesterol (27HC), may be important in breast cancer etiology and explain associations between obesity and postmenopausal breast cancer risk. Epidemiologic evidence for 27HC in breast cancer risk is limited, particularly in multiethnic populations.

**Methods:**

In a nested case–control study of 1470 breast cancer cases and 1470 matched controls within the Multiethnic Cohort Study, we examined associations of pre-diagnostic circulating 27HC with breast cancer risk among African American, Japanese American, Native Hawaiian, Latino, and non-Latino White postmenopausal females. We used multivariable logistic regression adjusted for age, education, parity, body mass index, and smoking status. Stratified analyses were conducted across racial and ethnic groups, hormone receptor (HR) status, and use of lipid-lowering drugs. We assessed interactions of 27HC with steroid hormones.

**Results:**

27HC levels were inversely related to breast cancer risk (odds ratio [OR] 0.80; 95% confidence interval [CI] 0.58, 1.12), but the association was not statistically significant in the full model. Directions of associations differed by racial and ethnic group. Results suggested an inverse association with HR-negative breast cancer (OR 0.46; 95% CI 0.20, 1.06). 27HC interacted with testosterone, but not estrone, on risk of breast cancer; 27HC was only inversely associated with risk among those with the highest levels of testosterone (OR 0.46; 95% CI 0.24, 0.86).

**Conclusion:**

This is the first US study to examine circulating 27HC and breast cancer risk and reports a weak inverse association that varies across racial and ethnic groups and testosterone level.

**Supplementary Information:**

The online version contains supplementary material available at 10.1186/s13058-023-01693-6.

## Background

Breast cancer is the most common cancer and the second leading cause of cancer death among US females [1]. Obesity and body fat distribution are important risk factors for breast cancer [2–6]. Dyslipidemia accompanies obesity, and adipose tissue is a key source of circulating estrogen after menopause [7, 8]. At the same time, observational studies have reported associations of low high-density lipoprotein cholesterol (HDL-C) levels, high total cholesterol levels, and high triglyceride levels with increased breast cancer risk, although with some variability across studies [9–15]. Thus, hypothesized mechanisms for the association between obesity and postmenopausal breast cancer implicate higher levels of cholesterol and estradiol.

27-Hydroxycholesterol (27HC) is a cholesterol metabolite, the most-abundant circulating oxysterol, and an endogenous selective estrogen receptor modulator (SERM) [16–21]. 27HC levels co-vary with cholesterol and hypercholesterolemia results in commensurately high levels of 27HC (82). In mouse models, 27HC has been shown to promote breast cancer formation and growth by binding to the estrogen receptor (ER) and to increase the metastatic potential of breast tumors through the activation of the liver X receptor (LXR) [18–21]. In breast cancer cell lines, the role of 27HC in cell growth was modulated by levels of exogenous estradiol, so the impact of 27HC on breast cancer development may vary with concomitant levels of steroid hormones [17]. Thus, while 27HC is a potential molecular target for breast cancer prevention [20, 22–24], there is a need for epidemiologic studies that assess the influence of 27HC on breast cancer risk, especially in the presence of steroid hormones that also modulate estrogen signaling [25–31].

There are substantial racial and ethnic disparities in breast cancer risk in the US; breast cancer incidence rates range from a high of 172.1 per 100,000 person years among Native Hawaiian to a low of 92.1 among Hispanic females [32, 33]. Additionally, total cholesterol levels vary by racial and ethnic group in the US from a high of 13.1% among non-Hispanic White, females to 9.2% among Hispanic females [34]. These national prevalence estimates, however, report Asian American and Pacific Islander groups as a single aggregate group, masking the prevalence of high cholesterol that has been observed to be as high as 48% among Native Hawaiian and Pacific Islander females [35]. Thus, racial and ethnic disparities in hypercholesterolemia appear to track with disparities in breast cancer risk. However, in 2019, a nested case–control study within the European EPIC-Heidelberg cohort showed that higher levels of circulating 27HC were associated with a decreased risk of postmenopausal breast cancer among 193 cases and 200 controls within a largely White, European study population [36] (relative risk = 0.56 95% CI 0.34, 0.95). Therefore, we hypothesized that associations between 27HC and breast cancer risk may differ across racial and ethnic groups and that these differences may shed light on racial and ethnic disparities in breast cancer risk.

To our knowledge, there is no epidemiologic study that has assessed the role of circulating 27HC and breast cancer risk across groups defined by race and ethnicity or examined the interaction of 27HC with lipid or steroid hormone levels. Thus, we conducted a nested case–control study within the Multiethnic Cohort to examine associations of pre-diagnostic, circulating 27HC with breast cancer risk among African American, Japanese American, Latino, Native Hawaiian, and non-Latino White females (1470 cases and 1470 controls). Our primary aim was to assess differences in associations across groups defined by race and ethnicity and to examine interactions of 27HC with lipids and steroid hormones. We also examined associations of 27HC with breast cancer according to hormone receptor (HR) status and use of lipid-lowering drugs. Our secondary aim was to assess differences in associations of lipids and steroid hormones with breast cancer risk across racial and ethnic groups.

## Methods

### Study population and outcome assessment

The Multiethnic Cohort (MEC) is a prospective cohort of 215,251 individuals (male and female) 45–75 years of age at enrollment between 1993 and 1996 [37]. Participants are from five racial and ethnic groups categorized as African American, Japanese American, Latino, Native Hawaiian, and non-Latino White and live in Hawaiʻi or California (primarily Los Angeles County). Participants completed a baseline questionnaire that assessed demographics, lifestyle, diet, and anthropometrics, and for females, menstrual and reproductive history, and hormone therapy use. In 2001–2006, a prospective biorepository was established that collected blood specimens from approximately 70,000 healthy participants. A short questionnaire was administered at biospecimen collection that assessed age, smoking status, weight, and hormone therapy use. In 2003–2008, a follow-up questionnaire was administered that ascertained information on use of lipid-lowering drugs.

Participants diagnosed with invasive breast cancer were identified through linkage to the Hawaiʻi and California tumor registries of the Surveillance, Epidemiology, and End Results Program of the National Cancer Institute. Case and control selection are illustrated in Additional file 1: Fig. 1. This nested case–control study included 1470 females diagnosed with incident invasive breast cancer who were postmenopausal or age > 50 years at cohort entry and provided sufficient pre-diagnostic blood (plasma) sample (cases). Controls were female participants who provided blood samples and were alive and breast cancer free at the age of the matched case’s diagnosis. Controls were matched 1:1 to cases on geographic area (Hawaiʻi or California), race and ethnicity (African American, Japanese American, Latino, Native Hawaiian, and non-Latino White), age at blood draw (± 1 year), birth year (± 1 year), time of blood draw (± 2 h), and hours fasting prior to blood draw (groups: 0 to < 6, 6 to  < 8, 8 to  < 10, 10 + hours). Cases that did not match with these criteria (*n* = 192, 15%) were matched by sequentially relaxing criteria to comprise age at blood draw and birth year ± 3 years (6.6%), time of blood draw ± 4 h and < / = 1 adjacent fasting group (4.8%), age at blood draw and birth year ± 5 years (3.0%), time of blood draw ± 6 years and < / = 2 fasting groups adjacent (0.4%), and finally, time of blood draw ± 8 years and < / = 4 fasting groups adjacent (0.2%). Thus, the final study population consisted of 1470 matched pairs.

### Exposures of interest—blood plasma analytes

Our main exposure of interest was circulating 27HC. We also assessed circulating lipids (i.e., high-density lipoprotein cholesterol [HDL-C], low-density lipoprotein cholesterol [LDL-C], total cholesterol, and triglycerides), steroid hormones (i.e., total estradiol, estrone, and testosterone), and sex hormone binding globulin (SHBG).

Biomarker assays were conducted at the University of Hawaiʻi Cancer Center Analytical Biochemistry Shared Resource under the supervision of Dr. Adrian Franke. Plasma samples were prepared according to Honda et al. with slight modifications [38] (Additional file 1). 27HC and steroid hormone levels were measured with liquid chromatography-orbitrap mass spectrometry (LC/MS) (Additional file 1) [39]. One matched pair was missing for steroid biomarker assays. Lipid assays were conducted as part of a previous study, so not all cases had lipid assay measurements available (4 of 1470 matched pairs missing). HDL-C, total cholesterol, and triglyceride levels were assayed by an automated chemical analyzer (Cobas Mira Plus, Roche Diagnostics, Switzerland). LDL-C was derived by applying the Friedewald formula, except for participants whose fasting triglyceride was over 400 mg/dl, which resulted in exclusion of an additional 11 matched pairs for LDL-C [40, 41]. Plasma SHBG was assayed by double-antibody enzyme-linked immunosorbent-assay according to manufacturer’s specifications (R&D Systems, Minneapolis, MN) [42].

Laboratory personnel were blinded to case–control status. Matched pairs were assayed in the same batch; quality control pairs were included within each batch, and duplicate pairs were included between batches. All coefficients of variation (CVs) are available in Additional file 1; within-batch CVs were all < 7%, except for estradiol and testosterone which were 16% and 14%, respectively. Analytes below the lower limit of detection (LLOD) were assigned a value of half of the LLOD. For 27HC, no samples were below LLOD. For estradiol, 49.9% of control and 44.7% of case samples were below LLOD. For estrone, 0.4% of control and 0.2% of case samples were below LLOD. For testosterone, 0.4% of control and 0.3% of case samples were below LLOD.

### Covariates

Covariates included socioeconomic, behavioral, and reproductive factors assessed at baseline or blood collection and consisted of factors that were hypothesized confounders of associations between 27HC and breast cancer risk or previously observed to be associated with breast cancer risk (Additional file 1: Fig. 2). Hypothesized confounders were age at blood draw, education, body size, smoking status, alcohol, and diet. Reproductive factors previously observed to be associated with breast cancer risk (i.e., parity, age at menarche, oral contraception, and hormone replacement therapy) were considered since inclusion of these covariates if associated with breast cancer risk in our sample would confer increased precision in our estimates.

Baseline measures were individual-level education (less than high school, some college, college graduate, graduate school, missing), alcohol consumption (yes, no, missing), Alternate Mediterranean Diet total score [43] (quartiles based on distribution of scores in the study population), age at menarche (≤ 12 years, 13–14 years, > 14 years, missing), parity (0,1,2–3, ≥ 4, missing), oral contraceptive use (yes, no), and hormone replacement therapy (never estrogen, past estrogen, current estrogen without progesterone, current estrogen with progesterone, missing).

Covariates collected at the time of blood collection were age at blood draw, body mass index (BMI; underweight and normal weight, < 25.0; overweight, 25.0–29.9; and obese, > 30.0 kg/m^2^, missing), smoking status and pack-years (never smoker, former smoker/ < 20 pack-years, former smoker/ ≥ 20 pack-years, current smoker/ < 20 pack-years, current smoker/ ≥ 20 pack-years, missing), hormone replacement therapy (yes, no, missing), and lipid-lowering drug use (yes, no, missing).

For breast cancer cases, hormone receptor (HR) status was obtained from cancer registries (HR-positive, estrogen receptor- [ER] or progesterone receptor- [PR] positive; HR-negative, ER- and PR-negative, missing).

### Statistical analysis

We calculated the mean, SD, and range of each analyte among control and case populations in the overall study population and across racial and ethnic groups. Distributions of raw values of biomarkers were right skewed; thus, the log-transformed values of biomarkers were used for analysis. We generated frequency distributions of race and ethnicity, HR status, lipid-lowering drug use, and covariates among cases and controls.

We used the quasi-Newton method to determine optimal parameters for multivariable conditional logistic regression to estimate odds ratios (ORs) and 95% confidence intervals (CIs) for associations between log-transformed levels of circulating analytes and breast cancer risk [44]. We examined each circulating analyte in the overall study population and according to race and ethnicity, HR status, and lipid-lowering drug use. 27HC was also analyzed according to levels of testosterone (described below). The frequencies of matched pairs that have the same category of stratifying variables within each model are noted in figures.

Potential covariates were determined with a directed acyclic graph (DAG) involving 27HC, lipid levels, and steroid hormones to breast cancer diagnosis (Additional file 1: Fig. 2). Minimal models were adjusted for age at blood draw. We tested associations between each potential covariate with breast cancer diagnosis; potential covariates with type 3 *P*-values < 0.1 (Additional file 1: Table 1) were included in fully adjusted models. Full models were adjusted for baseline education and parity as well as age, BMI, and smoking status at blood draw. Spline models with tests for polynomial forms did not indicate substantial nonlinearity, so we present linear model forms with analytes of interest modeled as continuous. ORs represent the increase in odds of breast cancer diagnosis for every unit increase in log-transformed analyte concentration.

To assess joint effects between 27HC and other analytes, an interaction term for 27HC and each lipid, steroid hormone, and SHBG were assessed separately in the full model. Using the Wald test, only the model with the interaction term for 27HC and testosterone levels was significant at *p* < 0.05 (*p* for interaction = 0.013). Thus, the association between 27HC and breast cancer risk was assessed across four quartiles of testosterone based on levels among controls (quartile 1, 5 to < 72.5; quartile 2, 72.5 to < 105.6; quartile 3, 105.6 to < 152.5; quartile 4, ≥ 152.46 mg/dL). Only matched pairs with testosterone levels within the same quartile of testosterone were used in this analysis.

To assess whether 27HC associations with breast cancer risk were independent of steroid hormones, full models were additionally adjusted for estrone and testosterone. We also assessed whether associations between 27HC and breast cancer diagnosis differed by BMI status (low BMI, underweight/normal; high BMI, overweight/obese).

To summarize our results on the association between 27HC and breast cancer risk with those of the mostly White, European EPIC-Heidelberg Cohort [36], we used random effects meta-analysis to calculate pooled ORs from study-specific ORs (this study) or relative risks (EPIC-Heidelberg) available for (1) the total study population of this study with postmenopausal females of the EPIC-Heidelberg study, (2) the White female population of this study with postmenopausal females of the EPIC-Heidelberg study, and (3) HR-positive breast cancer. Forest plots were generated to visualize study-specific and pooled estimates.

All *p*-values were two-sided; *p*-values < 0.05 were considered statistically significant. All analyses except for meta-analyses were conducted using SAS version 9.4 (SAS Institute, Cary, NC). Meta-analyses were conducted with Stata v17 (StataCorp, College Station, TX).

## Results

Levels of 27HC, lipids, steroid hormones, and SHBG among controls and cases are presented in Table 1. Mean 27HC was 83.9 pg/mL (SD, 22.5 pg/mL) among controls and 82.6 pg/mL (SD, 21.58) among cases. Table 2 shows levels of 27HC across racial and ethnic groups; African American and Native Hawaiian females (both cases and controls) had higher 27HC levels than the mean among the overall study sample. Additional file 1: Table 2 provides Spearman correlation coefficients for biomarkers. 27HC was weakly correlated with total cholesterol (*ρ* = 0.14) and triglycerides (*ρ* = 0.28). Given that estrone and estradiol were correlated (*ρ* = 0.72) and over 45% of estradiol observations were below the LLOD, while less than 0.5% of estrone observations were below the LLOD, we restricted further analysis to the estrone biomarker. Frequency distributions of subgroups are presented in Table 3. One-third (35%) of the study population were Japanese American, 19% were non-Latino White, 19% were Latino, 15% were African American, and 12% were Native Hawaiian.

**Table 1:** Circulating levels of biomarkers of interest according to case–control status among postmenopausal females, the Multiethnic Cohort

Biomarkers of interest	Matched pairs	Controls				Breast cancer cases			
	*N*	Mean	SD	Min	Max	Mean	SD	Min	Max
27HC (ng/mL)	1470	83.89	22.45	33.08	261.9	82.63	21.58	0.57	200.46
*Lipids*^***a***^									
HDL-C (mg/dL)	1466	51.96	19.04	1.00	146	44.49	17.17	2.64	131.05
LDL-C (mg/dL)	1455	131.96	39.25	25.20	346.4	135.31	37.95	25.00	307.20
Triglycerides (mg/dL)	1466	120.46	73.27	20.00	1000	118.84	79.41	21.00	995.00
Total cholesterol (mg/dL)	1466	207.69	41.68	49.70	500	203.58	38.79	77.00	438.14
*Steroid hormones*^b^									
Estrone (pg/mL)	1469	563.16	1472.85	2.50	27,713	633.25	1477.97	2.50	19,708.89
Testosterone (pg/mL)	1469	129.78	171.65	5.00	5300.3	150.19	240.96	5.00	8056.94
SHBG (nM)	1470	77.13	53.51	6.36	404	68.23	51.01	6.00	404.00

^a^Lipid assays were conducted as part of a previous study, so not all cases had lipid assay measurements available (4 of 1470 matched pairs missing). An additional 11 matched pairs are missing for LDL-C, because LDL-C levels are derived from fasting triglyceride levels and samples with fasting triglyceride levels over 400 mg/dl were excluded from this derivation procedure

^b^One sample missing from steroid hormone assays

*27HC* 27-hydroxy cholesterol, *HDL-C* high-density lipoprotein cholesterol, *LDL-C* low-density lipoprotein cholesterol (calculated), *SHBG* sex hormone binding globulin, *SD* standard deviation

**Table 2:** Circulating levels of 27HC according to case–control status and racial and ethnic group among postmenopausal females, the Multiethnic Cohort

27HC (ng/mL)	Matched pairs	Controls				Breast cancer cases			
	*N*	Mean	SD	Min	Max	Mean	SD	Min	Max
Overall	1470	83.89	22.45	33.08	261.90	82.63	21.58	0.57	200.46
*Race and ethnicity*									
African American	220	85.64	23.91	33.08	171.71	86.37	22.17	36.70	156.67
Japanese American	517	83.53	22.01	36.73	203.88	81.74	20.67	0.57	166.97
Latino	272	82.68	20.88	33.29	175.09	80.04	21.61	31.76	200.46
Native Hawaiian	177	86.30	23.19	43.58	261.90	84.26	22.78	36.20	188.00
Non-Latino White	284	82.86	23.03	35.21	236.53	82.83	21.60	33.18	168.26

*27HC* 27-hydroxycholesterol, *SD* standard deviation

**Table 3:** Frequency distribution of covariates among the study population according to case–control status among postmenopausal females, the Multiethnic Cohort

	Total (*N* = 2940)	Controls (*N* = 1470)	Breast cancer cases
	*N* (%)	*N* (%)	*N* (%)
Race and ethnicity			
African American	440 (15.0%)	220 (15.0%)	220 (15.0%)
Japanese American	1034 (35.2%)	517 (35.2%)	517 (35.2%)
Latino	544 (18.5%)	272 (18.5%)	272 (18.5%)
Native Hawaiian	354 (12.0%)	177 (12.0%)	177 (12.0%)
Non-Latino White	568 (19.3%)	284 (19.3%)	284 (19.3%)
HR status			
HR + (ER + or PR +)	2410 (82.0%)	1205 (82.0%)	1205 (82.0%)
HR− (ER− and PR− )	434 (14.8%)	217 (14.8%)	217 (14.8%)
Lipid-lowering drug use			
No	2070 (70.4%)	1060 (72.1%)	1010 (68.7%)
Yes	854 (29.0%)	404 (27.5%)	450 (30.6%)
Age at blood draw, mean years (SD)	66.4 (8.0)	66.4 (7.9)	66.3 (8.0)
Education			
< = High school	980 (33.3%)	462 (31.4%)	518 (35.2%)
Some college	939 (31.9%)	470 (32.0%)	469 (31.9%)
College graduate	493 (16.8%)	254 (17.3%)	239 (16.3%)
Graduate school	512 (17.4%)	276 (18.8%)	236 (16.1%)
Missing	16 (0.5%)	8 (0.5%)	8 (0.5%)
Parity			
None	351 (11.9%)	152 (10.3%)	199 (13.5%)
1 child	310 (10.5%)	161 (11.0%)	149 (10.1%)
2–3 children	1449 (49.3%)	746 (50.7%)	703 (47.8%)
4 + children	813 (27.7%)	402 (27.3%)	411 (28.0%)
Missing	17 (0.6%)	9 (0.6%)	8 (0.5%)
BMI at blood draw, kg/m^2^			
Underweight/normal	1140 (38.8%)	641 (43.6%)	499 (33.9%)
Overweight	990 (33.7%)	479 (32.6%)	511 (34.8%)
Obese	796 (27.1%)	347 (23.6%)	449 (30.5%)
Missing	14 (0.5%)	3 (0.2%)	11 (0.7%)
Smoking status at blood draw			
Never smoking	1728 (58.8%)	918 (62.4%)	810 (55.1%)
Former smoker	1044 (35.5%)	492 (33.5%)	552 (37.6%)
Current smoker	165 (5.6%)	57 (3.9%)	108 (7.3%)
Missing	3 (0.1%)	3 (0.2%)	0 (0.0%)

**Fig. 1:**
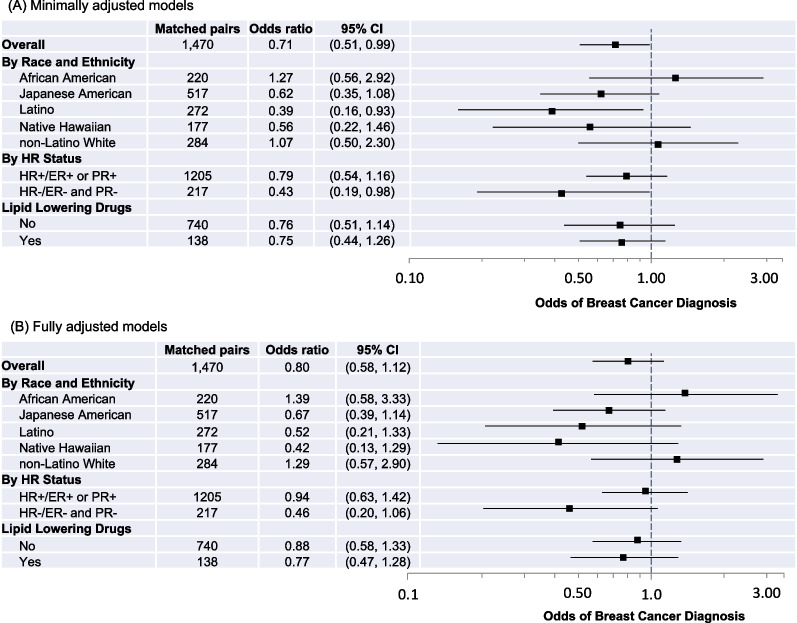
Odds ratios and 95% confidence intervals (CIs) for associations between 27HC (continuous) and breast cancer diagnosis overall and according to joint race and ethnicity, HR status, and lipid-lowering drug use. **A** Minimally adjusted models are adjusted for age at blood draw. **B** Fully adjusted models are adjusted for age at blood draw, education, parity, BMI at blood draw, and smoking status at blood draw. Odds ratios are indicated with black squares; 95% CIs are indicated with solid lines; the x-axis is on the log scale. For each model, controls are the reference group; the reference odds ratio (1.0) is indicated by the vertical dashed line

Results from minimally adjusted models are presented in Fig. 1A; 27HC was significantly inversely associated with risk among the overall population (OR 0.71; 95% CI 0.51–0.99). In analyses stratified by racial and ethnic group, 27HC was inversely associated with risk among Latino females (OR 0.39; 95% CI 0.16–0.93) and Japanese American females, although the estimate among Japanese American females was not statistically significant (OR 0.62; 95% CI 0.35–1.08). 27HC was inversely associated with risk among those with HR-negative breast cancer (OR 0.43; 95% CI 0.19–0.98) but not HR-positive breast cancer. Of potential covariates (see DAG in Additional file 1: Fig. 2), education, BMI, parity, and smoking status were associated with breast cancer risk at *p* < 0.1 (Additional file 1: Table 1), so these covariates were included in fully adjusted models.

Results from fully adjusted models of the association between 27HC and breast cancer risk are presented in Fig. 1B. There were no statistically significant associations among the overall population or in stratified analyses. However, 27HC may be inversely associated with risk for certain groups; effect sizes among Japanese American, Latino, and Native Hawaiian females suggest weak inverse associations (Japanese American, OR 0.67, 95% CI 0.39–1.14; Latino, OR 0.52, 95% CI 0.21–1.33; Native Hawaiian, OR 0.42, 95% CI 0.13–1.29). A test for heterogeneity across racial and ethnic groups was null (*p* = 0.26). Results also suggest a weak inverse association of 27HC for HR-negative breast cancer (HR- OR 0.46, 95% CI 0.20–1.06). The *p*-value for a test of heterogeneity across HR status was *p* = 0.14.

**Fig. 2:**
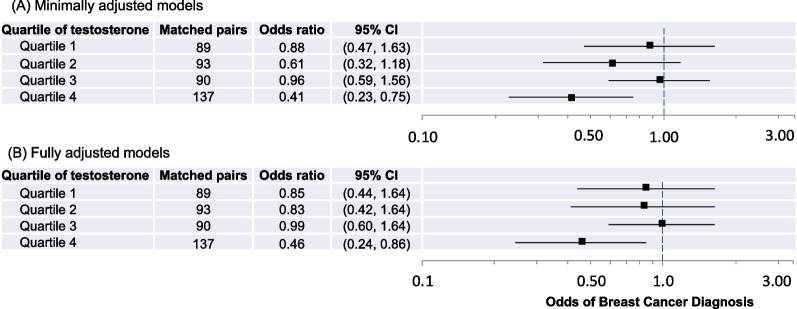
Odds ratios and 95% confidence intervals (CIs) for associations between 27HC and breast cancer diagnosis according to quartile of testosterone level. **A** Minimally adjusted models are adjusted for age at blood draw. **B** Fully adjusted models are adjusted for age at blood draw, education, parity, BMI at blood draw, and smoking status at blood draw. Odds ratios are indicated with black squares; 95% CIs are indicated with solid lines; the x-axis is on the log scale. For each model, controls are the reference group; the reference odds ratio (1.0) is indicated by the vertical dashed line

We observed a significant interaction between 27HC and testosterone levels (*p* = 0.013). Thus, we examined associations between 27HC and breast cancer risk in the overall study population across quartiles of testosterone (Fig. 2). 27HC was associated with breast cancer risk only among females with the highest levels of testosterone (OR 0.46, 95% CI 0.24–0.86). A test for trend was null (*p*-trend = 0.22). We did not observe interactions between 27HC and estrone, SHBG, or lipid biomarkers.

**Fig. 3:**
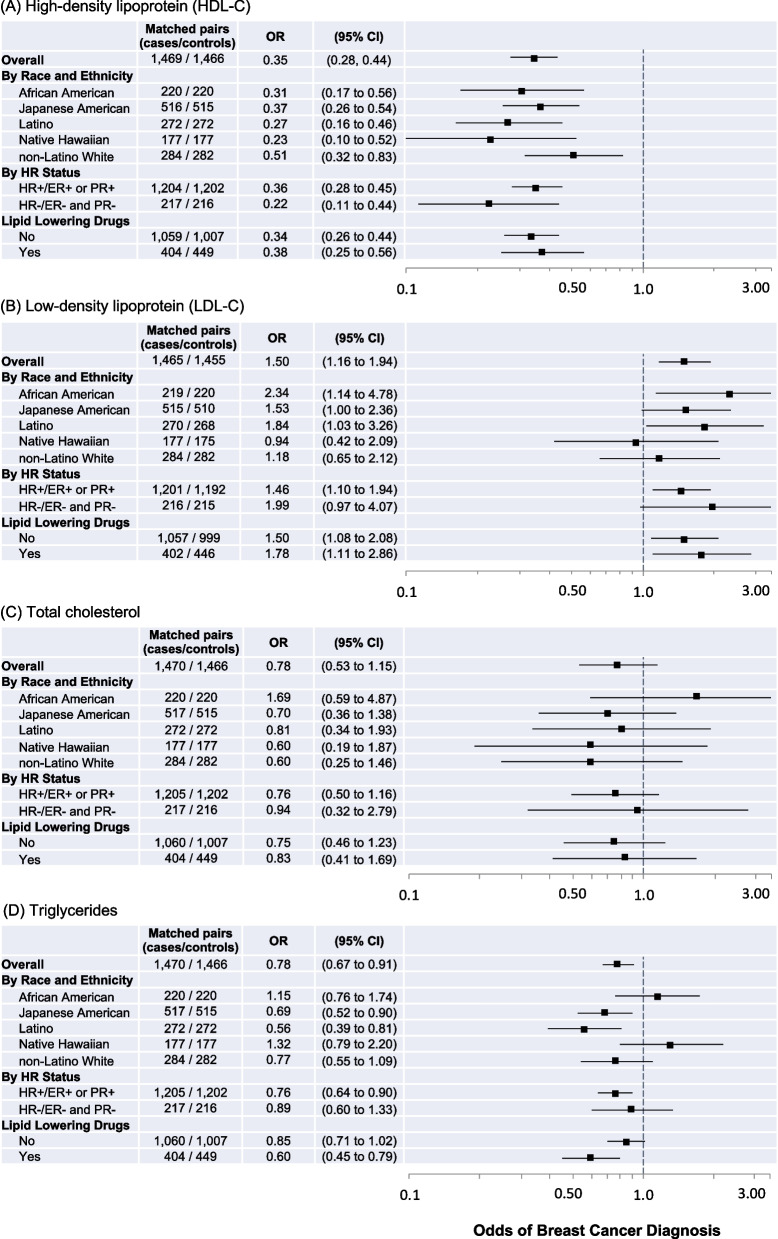
Fully adjusted models estimating odds ratios and 95% confidence intervals (CIs) of breast cancer diagnosis associated with **A** high-density lipoprotein (HDL-C), **B** low-density lipoprotein (LDL-C), **C** total cholesterol, and **D** triglyceride levels overall and according to joint race/ethnicity, HR status, and lipid-lowering drug use. All models are adjusted for age at blood draw, education, parity, BMI at blood draw, and smoking status at blood draw. Odds ratios are indicated with black squares, 95% CIs indicated with solid lines; the x-axis is on the log scale. For each model, controls are the reference group; the reference odds ratio (1.0) is indicated by the vertical dashed line

Figure 3 shows results from fully adjusted models of associations between lipids and breast cancer risk. HDL-C was strongly inversely associated with breast cancer risk among the overall study population (OR 0.35, 95% CI 0.28–0.44) and across all groups in stratified analyses. LDL-C was associated with higher breast cancer risk among the overall study population (OR 1.50, 95% CI 1.16–1.94), but effect sizes differed across racial and ethnic groups. For total cholesterol, an inverse association among the overall study population was suggested but not statistically significant (OR 0.78, 95% CI 0.53–1.15); African American females were the only racial and ethnic group for which this direction of association for total cholesterol was positive (OR 1.69, 95% CI 0.59–4.87). Triglyceride levels were inversely associated with risk among the overall study population (OR 0.78, 95% CI 0.67–0.91), for Japanese American and Latino females, and for HR-positive breast cancer. Among lipids, only associations between triglyceride and breast cancer risk had significant statistical heterogeneity across racial and ethnic groups (*p* = 0.02) and for lipid-lowering drug use (*p* = 0.02).

Additional file 1: Table 3 shows results from fully adjusted models of associations of steroid hormones and SHBG with breast cancer diagnosis. Estrone level was positively associated with breast cancer risk among the overall study population (OR 1.13, 95% CI 1.07–1.21), but associations varied across racial and ethnic group. Testosterone level was positively associated with risk among the overall study population (OR 1.39, 95% CI 1.22–1.58) as well as for the African American (OR 1.57, 95% CI 1.13–2.17), the Japanese American (OR 1.66, 95% CI 1.31–2.09), and the non-Latino White groups (OR 1.28, 95% CI 0.96–1.71) and for HR-positive breast cancers (OR 1.44, 95% CI 1.25–1.67). SHBG levels were inversely associated overall (OR 0.75, 95% CI 0.66–0.86) and generally across all groups in stratified analyses, although not all estimates are statistically significant.

Estimates of associations between 27HC and breast cancer risk did not change with further adjustment for levels of estrone and testosterone (Additional file 1: Table 4). While not statistically significant, the inverse association between 27HC and breast cancer risk may be specific to those with a BMI status of underweight/normal (OR 0.65, 95% CI 0.40, 1.06) (Additional file 1: Table 5).

The overall odds ratio summarizing results from the Multiethnic Cohort and the postmenopausal population of the EPIC-Heidelberg cohort indicates a significant inverse association between 27HC and breast cancer risk (summary OR 0.72, 95% CI 0.54–0.94), with a similar but not statistically significant association among non-Latino White females within the MEC and postmenopausal females within EPIC-Heidelberg (summary OR 0.70, 95% CI 0.46–1.06) and among HR-positive cases within the Multiethnic Cohort and ER-positive postmenopausal cases within EPIC-Heidelberg (summary OR 0.83, 95% CI 0.60–1.16) (Additional file 1: Fig. 3).

## Discussion

Our study of the Multiethnic Cohort is the first in the US to examine the association between circulating 27HC and breast cancer risk and suggests an inverse association between 27HC and postmenopausal breast cancer risk that varies across racial and ethnic groups. This inverse association also seems to be restricted to HR-negative breast cancer in fully adjusted models. In addition, the inverse association of 27HC with breast cancer risk is apparent only among those with the highest levels of testosterone. Associations of 27HC with breast cancer risk were otherwise independent of steroid hormone and lipid levels.

A 2019 study of circulating 27HC and breast cancer risk among the EPIC-Heidelberg Cohort reported an inverse association between 27HC levels and breast cancer risk among postmenopausal females within the predominantly White, European cohort (193 cases, 200 controls) [36]. Results from our study of 1470 cases and 1470 controls in the Multiethnic Cohort also suggest an inverse association for 27HC but indicate that results from the EPIC-Heidelberg Cohort may not be generalizable to a multiracial and multiethnic US population. In fact, we found a positive, but not-statistically significant, association between 27HC levels and breast cancer risk among non-Latino White females (284 matched pairs), the group presumably most like the EPIC-Heidelberg Cohort in terms of self-identified race and ethnicity. A non-significant summary OR was observed in a meta-analysis comparing results from this population with results among postmenopausal females of the EPIC-Heidelberg study.

Notably, our results did not confirm those from animal studies that suggest 27HC would confer increased risk for breast cancer [18–21]. It is likely that multilevel influences on lipid and steroid hormone levels and complex functional interactions among lipids and steroid hormones require larger prospective studies with deep exposure assessment. This includes assessing whether the inverse association of 27HC with breast cancer is dependent on exposures or factors not measured in the current study. Moreover, even if 27HC levels do not confer observable risk to breast cancer formation, it is possible that, after cancer formation, 27HC levels could, as animal studies suggest, promote tumor growth and metastasis and thus worse prognosis. Future studies should examine this possibility.

While most of our estimates of the association between 27HC and breast cancer risk across racial and ethnic groups were not statistically significant, they do suggest differences in the association between 27HC and risk across racial and ethnic groups. Specifically, higher levels of 27HC may be inversely associated with breast cancer risk among Japanese American, Latino, and Native Hawaiian females and positively associated among White and African American postmenopausal females. However, while this suggests that our hypothesis that associations would vary across racial and ethnic groups may be valid, our hypothesis that high 27HC levels would shed light on racial and ethnic disparities in breast cancer risk is not supported by our results. For example, 27HC is inversely associated with risk among Native Hawaiian females who have the highest breast cancer incidence rate as well as Latino females who have the lowest breast cancer incidence rate. Given the complexity of endocrine signaling, it may be that the influence of 27HC on breast cancer development may be modified by other endocrine molecules; our observation of an interaction between 27HC and testosterone in breast cancer risk supports this possibility.

Testosterone levels have been linked to higher breast cancer risk [25, 26, 28–30, 45]; testosterone may promote cancer cell proliferation directly or by increasing estrogen levels. We also observed associations between higher testosterone levels and breast cancer. In addition, we observed an inverse association between 27HC and breast cancer risk only among individuals with the highest levels of testosterone, suggesting potential interaction between testosterone and 27HC. There is evidence that 27HC can mediate androgen receptor (AR) transcriptional activity and may facilitate ER and AR crosstalk activity [31]. However, the mechanism underlying testosterone modification of 27HC as a protective factor for breast cancer remains to be elucidated. Testosterone levels were also positively associated with HR-positive breast cancer [46], but we had insufficient sample sizes to stratify 27HC analyses simultaneously by race and ethnicity, HR subtype, and testosterone levels to further interrogate these interactions.

A 2015 meta-analysis of prospective cohort studies reported an inverse association between HDL-C and risk among postmenopausal females (RR = 0.45; 0.64, 0.93); these studies included only White females [47]. Our study of the racially and ethnically diverse Multiethnic Cohort confirms a consistent inverse association between prospective HDL-C and breast cancer. The same 2015 meta-analysis reported null results for total cholesterol with substantial heterogeneity across available estimates; our estimate of the association between total cholesterol and risk was non-significant but suggested an inverse association that was consistent across racial and ethnic groups except for African American females. Thus, our results are consistent with prior cohort studies to the extent that our study population can be compared with those of prior studies.

Notably, ours is the first study of 27HC among a multiracial, multiethnic population and thereby sheds light on differences in the mechanism of the impact of cholesterol on breast cancer risk across groups. There has only been one prospective cohort study among African American females that examined total cholesterol and found no association, but that study included females across a wide age range and used self-reported total cholesterol (high, not high) [48]. On the other hand, a 2013 case–control study of African American females in Washington DC reported a positive association of total cholesterol and LDL-C with breast cancer risk [49]. In our study, African American females were the only racial and ethnic group for which a positive association for total cholesterol was suggested and one of the groups for which a positive association for LDL-C was observed. Prospective cohort studies of lipids and breast cancer risk among postmenopausal Japanese American, Native Hawaiian, or Latino females are not, to our knowledge, available. Overall, our observations illustrate that future studies of the impact of cholesterol and cholesterol metabolites on breast cancer risk should consider modification of these processes by race and ethnicity and that the inverse association observed in White study populations may not be generalizable [47].

Our study of breast cancer risk within the Multiethnic Cohort with prospective assessment of 27HC, lipids, and steroid hormones and information on other covariates (e.g., health behaviors, breast cancer risk factors) offers substantial data to evaluate hypotheses regarding 27HC as a risk factor for breast cancer. However, we note some limitations. Despite being the largest and most diverse cohort population in the US to date to examine 27HC and breast cancer risk, assessing the mechanisms behind associations between 27HC or lipid levels and breast cancer risk may require larger populations, particularly in consideration of groups for which these associations differ (i.e., racial and ethnic groups, lipid-lowering drug use, levels of steroid hormones) and in consideration of the potential for differences in risk across breast cancer subtypes.

## Conclusion

Ours is the first US study to examine associations between 27HC and breast cancer risk and suggests a weak inverse association that varied across groups within a multiethnic population. The inverse association of 27HC with breast cancer risk was strongest in females with the highest levels of testosterone. Results from this study extend our understanding of the role of lipids and their metabolites in breast cancer risk as well as racial and ethnic differences in risk of postmenopausal breast cancer.

### Supplementary Information

**Additional file 1**. Supplemental methods, tables, and figures.

## Data Availability

The Multiethnic Cohort investigators and institutions affirm their intention to share the research data consistent with all relevant NIH resource/data sharing policies. Data requests should be submitted through Multiethnic Cohort online data request system at https://www.uhcancercenter.org/for-researchers/mec-data-sharing. Further information is available from the corresponding author upon request.

## Abbreviations

27HC: 27-Hydroxycholesterol
AR: Androgen receptor
BMI: Body mass index
CI: Confidence interval
CV: Coefficient of variation
DAG: Directed acyclic graph
ER: Estrogen receptor
HDL-C: High-density lipoprotein cholesterol
HR: Hormone receptor
LDL-C: Low-density lipoprotein cholesterol
LXR: Liver X receptor
OR: Odds ratio
PR: Progesterone receptor
SD: Standard deviation
SERM: Selective estrogen receptor modulator
SHBG: Sex hormone binding globulin
US: United States
