# Supplementary material for: Circulating 27-hydroxycholesterol, lipids, and steroid hormones in breast cancer risk: a nested case–control study of the Multiethnic Cohort Study
Source: Breast Cancer Res. 2023 Aug 14;25:95. doi: 10.1186/s13058-023-01693-6 (PMC10424359; doi:10.1186/s13058-023-01693-6)

**Supplementary Material**

**Supplemental Figure 1**. Flowchart of nested case-control selection in the Multiethnic Cohort.

**Supplemental Methods:** Analysis of hydroxylated cholesterols in plasma by LCMS

**Supplemental Figure 2.** Directed Acyclic Graph showing putative relationships between biomarkers, covariates, and breast cancer outcome.

**Supplemental Table 1.** Minimal (age-adjusted) models for the association between all covariates and breast cancer risk

**Supplemental Table 2.** Spearman correlation coefficients assessing correlation among plasma analytes

**Supplemental Table 3.** Odds ratios (ORs) and 95% confidence intervals (CI) for associations of steroid hormones and sex hormone binding globulin (SHBG) with breast cancer risk

**Supplemental Table 4.** Odds ratios (ORs) and 95% confidence intervals (CI) for associations of 27HC and breast cancer diagnosis with further adjustment for estrone and testosterone levels, the Multiethnic Cohort

**Supplemental Table 5**. Odds ratios (ORs) and 95% confidence intervals (CI) for associations of 27HC and breast cancer diagnosis according to BMI status, the Multiethnic Cohort

**Supplemental Figure 3.** Summary odds ratios (ORs) and 95% confidence intervals for meta-analyses summarizing results from this study of the Multiethnic Cohort and the EPIC-Heidelberg study of 27HC and breast cancer risk.

**Supplemental Figure 1. Flow chart of case- control selection**

**
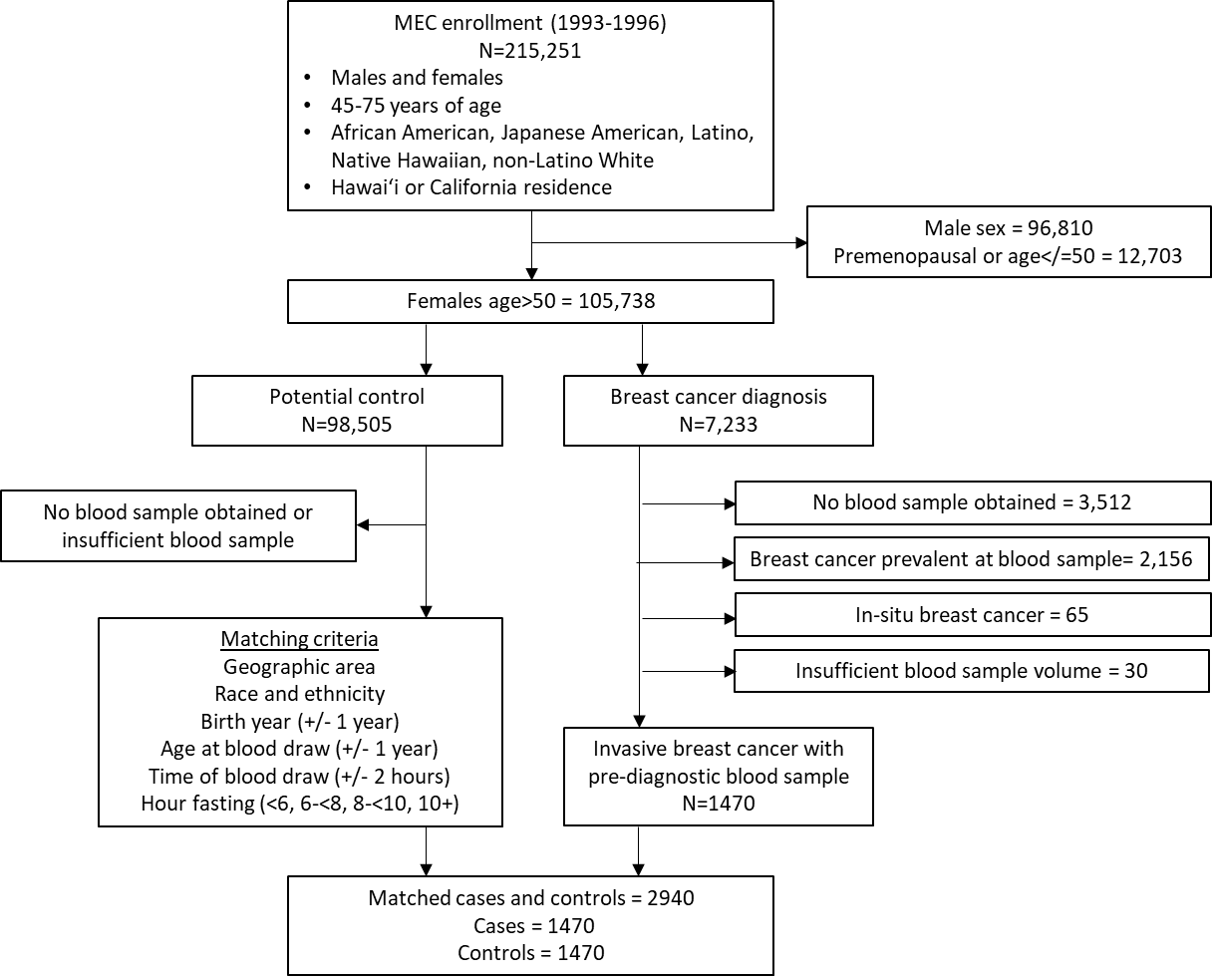
**

**Supplemental Methods.** Analysis of hydroxylated cholesterols in plasma by LCMS

**Sample preparation**

Plasma samples were prepared according to Honda et al (1) with slight modifications. In brief, 100 μl of plasma was diluted with 100 μl of ethanol and vortexed to mix. To the solution was added 20 μl of internal standard solution containing 24-hydroxycholesterol-d_7_, 27-hydroxycholesterol-d_5_, 25-hydroxycholesterol-d_3_ and progesterone-^13^C_2_ (1 μg/ml in methanol) and 50 μl of butylated hydroxytoluene (BHT, 1 mg/ml in ethanol). The resulting mixture was hydrolyzed with 200 μl of potassium hydroxide (1 M in ethanol) at 37 °C for 1 hr. After incubation, 1.2 ml of hexane and 200 μl of distilled water were added to the mixture consecutively. The hydrolyzed hydroxycholesterols were extracted to hexanes with vigorous vortexing, and the upper layer was aliquoted and dried under nitrogen in HPLC vials. The dried extract was then subjected to picolinyl derivatization. 150 μl of freshly prepared condensation mixture (containing 2-methyl-6-nitrobenzoic anhydride (27 mg), 4-dimethylaminopyridine (8 mg) picolinic acid (22 mg) in 8 ml anhydrous pyridine) and 20 μl of triethylamine were added into the dried sample, capped, vortexed to mix and heated at 65^o^C for 30 min. After incubation, the reaction mixture was dried under nitrogen, and redissolved in 100 μl acetonitrile and water (v/v 6/4).

**LCMS**

LCMS analysis was carried out on a model Accela ultra HPLC system coupled to a Q Exactive Orbitrap Mass Spectrometer and a CTC PAL autosampler (all from ThermoFisher, San Jose, CA). 20 μl of the above mixture was injected onto a Hypersil Gold C18 column (50 x 2.1 mm, 1.9 μm ThermoFisher, San Jose, CA) with a pre-column filter (0.2 µm, ThermoFisher, San Jose, CA). Gradient elution was performed at a flow rate of 400 μl /min using 0.1% formic acid in H_2_O (A), 0.1% formic acid in MeOH (B) and 0.1% formic acid in acetonitrile (C) as follows: 0-7.0 min linear gradient from 40%A to 6%A, 30%B to 47%B, 30%C to 47%C; hold at the same ratio for 8 min; then go back to first line condition and equilibrate for 5 minutes. Total HPLC time including equilibration was 15 minutes. Mass analysis was performed under positive electrospray target SIM mode, the conditions are as follows: (+) ESI spray voltage 4.5 kv, capillary transfer temperature 350°C, HESI heater temperature 350°C, sheath gas flow rate 35 unit; auxiliary gas 5 unit, in-source CID 5 ev, scan range 150 ~ 1000. AGC target 1e6, maximum injection time 100 ms, resolution 35,000, microscan 1. Quantitation of all analytes was performed with Xcalibur™ software by extracting the within 5 ppm of the calculated exact masses.

Mass list for quantification

| 24OH-cholesterol (2P+H) | 613.39998 |
| --- | --- |
| 24OH-cholesterol-d7 (2P+H) | 620.44392 |
| 27OH-cholesterol-d5 (2P+H) | 618.43137 |
| 25OH-cholesterol-d3 (2P+H) | 616.41882 |

**Coefficients of Variation**

The CV% (standard deviation (SD)/mean concentration x 100) within-batch was 3.8 for 27HC, 3.6 for HDL-C, 3.8 for total cholesterol, 3.8 for triglycerides, 15.9 for estradiol, 6.5 for estrone, 13.8 for testosterone, and 5.5 for SHBG. The CV% between batches was 15.4 for 27HC, 3.0 for HDL-C, 2.6 for total cholesterol, 1.7 for triglycerides, 38.7 for estradiol, 16.1 for estrone, 23.6 for testosterone, and 6.3 for SHBG.

References

1. Honda A, Yamashita K, Hara T*, et al.* Highly sensitive quantification of key regulatory oxysterols in biological samples by LC-ESI-MS/MS. *J Lipid Res* 2009;50(2):350-357.

**Supplemental Figure 2.** Directed Acyclic Graph showing putative relationships between biomarkers, covariates, and breast cancer outcome. Timing of data collection indicated. Covariates considered for multivariable models are indicated by blue boxes; asterisks highlight those that associated with breast cancer risk with a p-value <0.1 in minimal models and thus included in the full multivariable model. Our study population comprises only females who are postmenopausal or more than 50 years of age; thus, menopausal status does not appear in our model as a potential covariate.


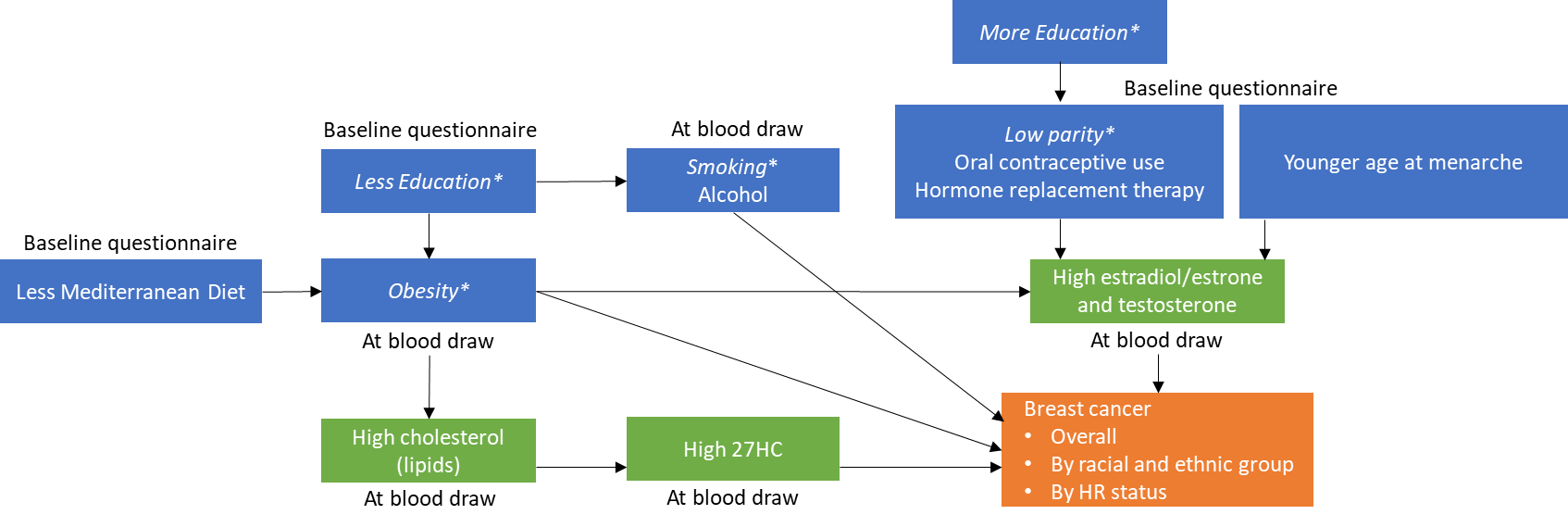


**Supplemental Table 1.** Minimal age-adjusted models^a^ for associations of all covariates (baseline, 1993-1996; blood draw, 2001-2006) with breast cancer risk among 1,470 cases and 1,470 controls.

| Variable | Cases  N (%) | Controls  N (%) | OR (95% CI)^e^ |
| --- | --- | --- | --- |
| Age at blood draw (continuous) | n/a | n/a | 1.25 (1.12 to 1.39) |
| Education at baseline |  |  |  |
| ≤ High school | 518 (35.2) | 462 (31.4) | 1.00 |
| Some college | 469 (31.9) | 470 (32.0) | 0.86 (0.71 to 1.04) |
| College graduated | 239 (16.3) | 254 (17.3) | 0.80 (0.63 to 1.01) |
| Graduate school | 236 (16.1) | 276 (18.8) | 0.72 (0.57 to 0.91) |
| Missing | 8 (0.5) | 8 (0.5) | 0.84 (0.31 to 2.25) |
| Type 3 P-value |  |  | 0.08 |
| Parity at baseline |  |  |  |
| None | 199 (13.5) | 152 (10.3) | 1.00 |
| 1 child | 149 (10.1) | 161 (11.0) | 0.70 (0.52 to 0.96) |
| 2-3 children | 703 (47.8) | 746 (50.7) | 0.72 (0.57 to 0.91) |
| 4+ children | 411 (28.0) | 402 (27.3) | 0.78 (0.60 to 1.01) |
| Missing | 8 (0.5) | 9 (0.6) | 0.69 (0.26 to 1.83) |
| Type 3 P-value |  |  | 0.09 |
| Age at Menarche at baseline |  |  |  |
| <12y | 794 (54.0) | 777 (52.9) | 1.00 |
| 13-14y | 516 (35.1) | 510 (34.7) | 0.99 (0.85 to 1.16) |
| >14y | 143 (9.7) | 167 (11.4) | 0.84 (0.66 to 1.07) |
| Missing | 17 (1.2) | 16 (1.1) | 1.05 (0.53 to 2.08) |
| Type 3 P-value |  |  | 0.55 |
| Menopausal status at baseline^b^ |  |  |  |
| Pre-Menopausal | 277 (18.8) | 296 (20.1) | 1.00 |
| Natural Menopause | 709 (48.2) | 674 (45.9) | 1.22 (0.94 to 1.58) |
| Surgical Menopause^b^ | 207 (14.1) | 218 (14.8) | 1.09 (0.81 to 1.46) |
| Other Surgery that causes periods to stop^c^ | 223 (15.2) | 226 (15.4) | 1.13 (0.84 to 1.51) |
| Period stopped but reason unknown^d^ | 39 (2.7) | 41 (2.8) | 1.09 (0.66 to 1.81) |
| Missing/Unknown | 15 (1.0) | 15 (1.0) | 1.11 (0.53 to 2.34) |
| Type 3 P-value |  |  | 0.55 |
| Smoking Status at blood draw |  |  |  |
| Non-smoker | 810 (55.1) | 918 (62.4) | 1.00 |
| Former smoker | 552 (37.6) | 492 (33.5) | 1.30 (1.11 to 1.52) |
| Current smoker | 108 (7.3) | 57 (3.9) | 2.31 (1.63 to 3.28) |
| Missing | 0 (0.0) | 3 (0.2) |  |
| Type 3 P-value |  |  | <.0001 |
| Alcohol Intake at baseline |  |  |  |
| Non-drinker | 825 (56.1) | 831 (56.5) | 1.00 |
| Drinker | 609 (41.4) | 602 (41.0) | 1.02 (0.88 to 1.19) |
| Missing | 36 (2.4) | 37 (2.5) | 0.98 (0.60 to 1.58) |
| Type 3 P-value |  |  | 0.96 |
| Alternate Mediterranean Diet Total Score at baseline |  |  |  |
| Quartile 1 (score 0-3) | 533 (36.3) | 505 (34.4) | 1.00 |
| Quartile 2 (score 4) | 287 (19.5) | 293 (19.9) | 0.92 (0.75 to 1.13) |
| Quartile 3 (score 5-6) | 481 (32.7) | 481 (32.7) | 0.95 (0.80 to 1.13) |
| Quartile 4 (score 7-9) | 133 (9.0) | 154 (10.5) | 0.82 (0.63 to 1.06) |
| Missing | 36 (2.4) | 37 (2.5) | 0.91 (0.56 to 1.49) |
| Type 3 P-value |  |  | 0.67 |
| BMI at baseline |  |  |  |
| Underweight/normal | 604 (41.1) | 751 (51.1) | 1.00 |
| Overweight | 529 (36.0) | 458 (31.2) | 1.47 (1.24 to 1.74) |
| Obese | 331 (22.5) | 259 (17.6) | 1.70 (1.38 to 2.11) |
| Missing | 6 (0.4) | 2 (0.1) | 3.57 (0.72 to 17.86) |
| Type 3 P-value |  |  | <.0001 |
| BMI at blood draw |  |  |  |
| Underweight/normal | 499 (33.9) | 641 (43.6) | 1.00 |
| Overweight | 511 (34.8) | 479 (32.6) | 1.41 (1.18 to 1.69) |
| Obese | 449 (30.5) | 347 (23.6) | 1.83 (1.49 to 2.24) |
| Missing | 11 (0.7) | 3 (0.2) | 4.46 (1.23 to 16.14) |
| Type 3 P-value |  |  | <.0001 |
| Oral Contraceptive at baseline |  |  |  |
| No | 1110 (75.5) | 1112 (75.6) | 1.00 |
| Yes | 360 (24.5) | 358 (24.4) | 1.01 (0.85 to 1.20) |
| Type 3 P-value |  |  | 0.93 |
| Hormone Replacement Therapy at baseline |  |  |  |
| Never estrogen (with or without progesterone) | 679 (46.2) | 704 (47.9) | 1.00 |
| Past estrogen (with or without progesterone) | 212 (14.4) | 195 (13.3) | 1.12 (0.90 to 1.41) |
| Current estrogen without progesterone | 194 (13.2) | 216 (14.7) | 0.93 (0.74 to 1.16) |
| Current estrogen with past or current progesterone | 337 (22.9) | 299 (20.3) | 1.17 (0.96 to 1.41) |
| Missing | 48 (3.3) | 56 (3.8) | 0.89 (0.59 to 1.32) |
| Type 3 P-value |  |  | 0.28 |
| Hormone Replacement Therapy at blood draw |  |  |  |
| No | 1009 (68.6) | 1033 (70.3) | 1.00 |
| Yes | 391 (26.6) | 378 (25.7) | 1.07 (0.90 to 1.26) |
| Unknown | 70 (4.8) | 59 (4.0) | 1.22 (0.85 to 1.75) |
| Type 3 P-value |  |  | 0.46 |

a. Each variable examined in a separate minimal model with conditional logistic regression adjusted for age at blood draw (continuous)

b. Oophorectomy with or without hysterectomy

c. Hysterectomy, Endometrial ablation

d. Including females greater than 65 years of age

e. OR = odds ratio; BMI = body mass index

**Supplemental Table 2.** Spearman correlation coefficients assessing correlation among plasma analytes

| Spearman Correlation Coefficient | 27HC | HDL | LDL | Total Cholesterol | Triglycerides | Estradiol | Estrone | Testosterone | SHBG |
| --- | --- | --- | --- | --- | --- | --- | --- | --- | --- |
| 27HC^a^ | 1 |  |  |  |  |  |  |  |  |
| HDL-C | -.02626 | 1 |  |  |  |  |  |  |  |
| LDL-C | .24835 | -.19348 | 1 |  |  |  |  |  |  |
| Total Cholesterol | .13592 | -.27346 | -.01619 | 1 |  |  |  |  |  |
| Triglycerides | .28184 | .15301 | .85728 | .15787 | 1 |  |  |  |  |
| Estradiol | -.01986 | .05686 | -.05108 | .03055 | -.00781 | 1 |  |  |  |
| Estrone | .02140 | .01766 | -.01890 | .05029 | .01228 | .71503 | 1 |  |  |
| Testosterone | .03106 | -.01262 | .04680 | -.07634 | .01109 | .20717 | .16173 | 1 |  |
| SHBG | -.02458 | .37522 | -.05682 | -.24991 | .04686 | .20102 | .09864 | .09399 | 1 |

a. 27HC = 27-hydroxy cholesterol; HDL-C = high-density lipoprotein cholesterol; LDL-C = low-density lipoprotein cholesterol; SHBG = sex hormone binding globulin

**Supplemental Table 3.** Odds ratios (ORs) and 95% confidence intervals (CI) for associations of steroid hormones and sex hormone binding globulin (SHBG) with breast cancer diagnosis^a^, the Multiethnic Cohort

|  | **Estrone** |  | **Testosterone** |  | **SHBG** |  |
| --- | --- | --- | --- | --- | --- | --- |
|  | **Cases/**  **Controls (N)** | **OR (95% CI)** | **Cases/**  **Controls (N)** | **OR (95% CI)** | **Cases/**  **Controls (N)** | **OR (95% CI)** |
| **Overall** | 1,469 / 1,469 | 1.13 (1.07 to 1.21) | 1,469 / 1,469 | 1.39 (1.22 to 1.58) | 1,470 / 1,470 | 0.75 (0.66 to 0.86) |
| **Race/Ethnicity** |  |  |  |  |  |  |
| African American | 219 / 220 | 1.20 (1.00 to 1.44) | 219 / 220 | 1.57 (1.13 to 2.17) | 220 / 220 | 0.60 (0.43 to 0.84) |
| Japanese American | 517 / 516 | 1.22 (1.10 to 1.35) | 517 / 516 | 1.66 (1.31 to 2.09) | 517 / 517 | 0.76 (0.60 to 0.95) |
| Latino | 272 / 272 | 0.98 (0.84 to 1.13) | 272 / 272 | 1.00 (0.75 to 1.32) | 272 / 272 | 0.79 (0.57 to 1.10) |
| Native Hawaiian | 177 / 177 | 1.06 (0.87 to 1.28) | 177 / 177 | 1.32 (0.85 to 2.03) | 177 / 177 | 0.85 (0.57 to 1.27) |
| White | 284 / 284 | 1.18 (1.03 to 1.37) | 284 / 284 | 1.28 (0.96 to 1.71) | 284 / 284 | 0.83 (0.61 to 1.13) |
| P for heterogeneity |  | 0.14 |  | 0.08 |  | 0.64 |
| **HR status** |  |  |  |  |  |  |
| HR+/ER+ or PR+ | 1,205 | 1.14 (1.06 to 1.22) | 1,205 | 1.44 (1.25 to 1.67) | 1,205 | 0.70 (0.61 to 0.82) |
| HR-/ER- and PR- | 216 | 1.12 (0.96 to 1.30) | 216 | 1.22 (0.85 to 1.75) | 217 | 0.94 (0.68 to 1.29) |
| P for heterogeneity |  | 0.8 |  | 0.40 |  | 0.11 |
| **Lipid lowering drug use** |  |  |  |  |  |  |
| No | 1,059 / 1,009 | 1.14 (1.06 to 1.23) | 1,059 / 1,009 | 1.27 (1.10 to 1.46) | 1,060 / 1,010 | 0.76 (0.65 to 0.89) |
| Yes | 404 / 450 | 1.12 (1.00 to 1.24) | 404 / 450 | 1.83 (1.43 to 2.34) | 404 / 450 | 0.73 (0.57 to 0.92) |
| P for heterogeneity |  | 0.56 |  | 0.004 |  | 0.61 |

a. All models are adjusted for age at blood draw, education, parity, BMI at blood draw, and smoking status at blood draw.

b. OR=odds ratio, CI=confidence interval, HR=hormone receptor status, ER=estrogen receptor, PR=progesterone receptor.

**Supplemental Table 4.** Odds ratios (ORs) and 95% confidence intervals (CI) for associations of 27HC and breast cancer diagnosis with further adjustment for estrone and testosterone levels^a^, the Multiethnic Cohort

|  | **OR** | **(95% CI)** |
| --- | --- | --- |
| **Overall** | 0.78 | (0.56, 1.10) |
| **Race/Ethnicity** |  |  |
| African American | 1.45 | (0.59, 3.57) |
| Japanese American | 0.63 | (0.36, 1.11) |
| Latino | 0.50 | (0.20, 1.29) |
| Native Hawaiian | 0.39 | (0.13, 1.23) |
| White | 1.31 | (0.57, 2.98) |
| **HR status** |  |  |
| HR+/ER+ or PR+ | 0.89 | (0.59, 1.34) |
| HR-/ER- and PR- | 0.50 | (0.22, 1.15) |
| **Lipid lowering drug use** |  |  |
| No | 0.86 | (0.56, 1.32) |
| Yes | 0.74 | (0.44, 1.26) |

a. All models are adjusted for age at blood draw, education, parity, BMI at blood draw, smoking status at blood draw, estrone levels and testosterone levels.

b. OR=odds ratio, CI=confidence interval, HR=hormone receptor status, ER=estrogen receptor, PR=progesterone receptor.

**Supplemental Table 5**. Odds ratios (ORs) and 95% confidence intervals (CI) for associations between 27HC and breast cancer diagnosis according to BMI status, the Multiethnic Cohort

| **BMI status^b^** | **Control N** | **Case N** | **Odds Ratio** | **(95% CI)** | **P-value** |
| --- | --- | --- | --- | --- | --- |
| Underweight/normal | 641 | 499 | 0.65 | (0.40, 1.06) | 0.08 |
| Overweight/Obese | 826 | 960 | 0.93 | (0.61, 1.43) | 0.74 |
| **P for heterogeneity** |  |  | . | . | 0.24 |

a. All models are adjusted for age at blood draw, education, parity, BMI at blood draw, smoking status at blood draw, estrone levels and testosterone levels.

b. BMI; underweight and normal weight, <25.0; overweight, 25.0-29.9; and obese, >30.0 kg/m^2^

c. OR=odds ratio, CI=confidence interval

**Supplemental Figure 3**. Study-specific and overall relative risk ratios and 95% confidence intervals for meta-analysis summarizing the association between circulating 27HC and breast cancer risk within the Multiethnic Cohort (MEC) and postmenopausal females of the EPIC-Heidelberg study (April 2019) for (A) the overall MEC study population and postmenopausal females of EPIC-Heidelberg, (B) non-Latino White females within the Multiethnic Cohort and the total postmenopausal EPIC-Heidelberg population, and (C) HR+ cases within the Multiethnic Cohort and ER+ breast cancer postmenopausal cases within the EPIC-Heidelberg study.

A. Overall MEC population, postmenopausal EPIC-Heidelberg


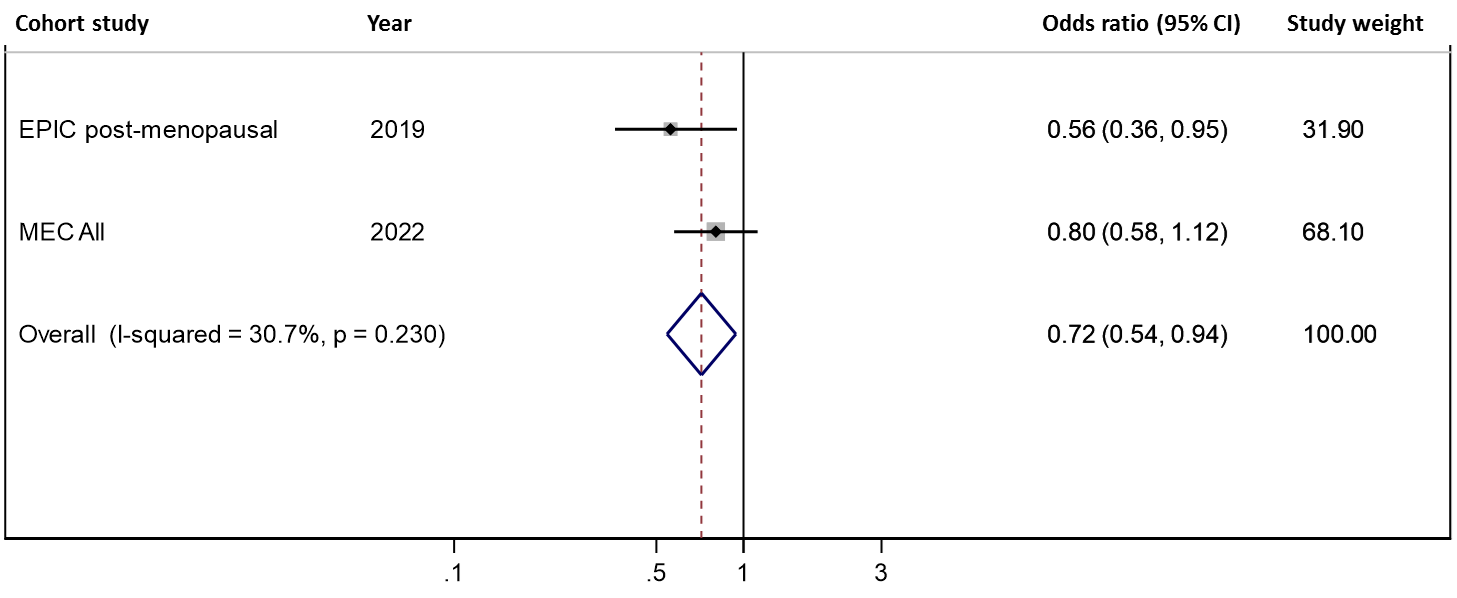


B. Multiethnic Cohort non-Latino White females, postmenopausal EPIC-Heidelberg


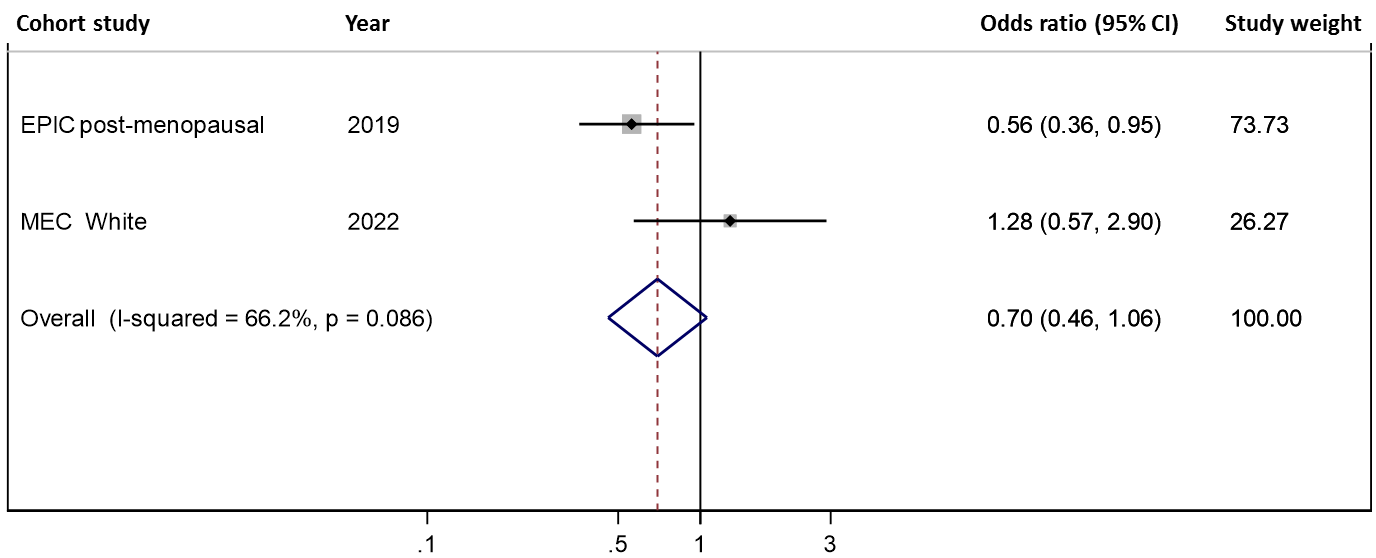


C. Multiethnic Cohort HR+, postmenopausal EPIC-Heidelberg ER+


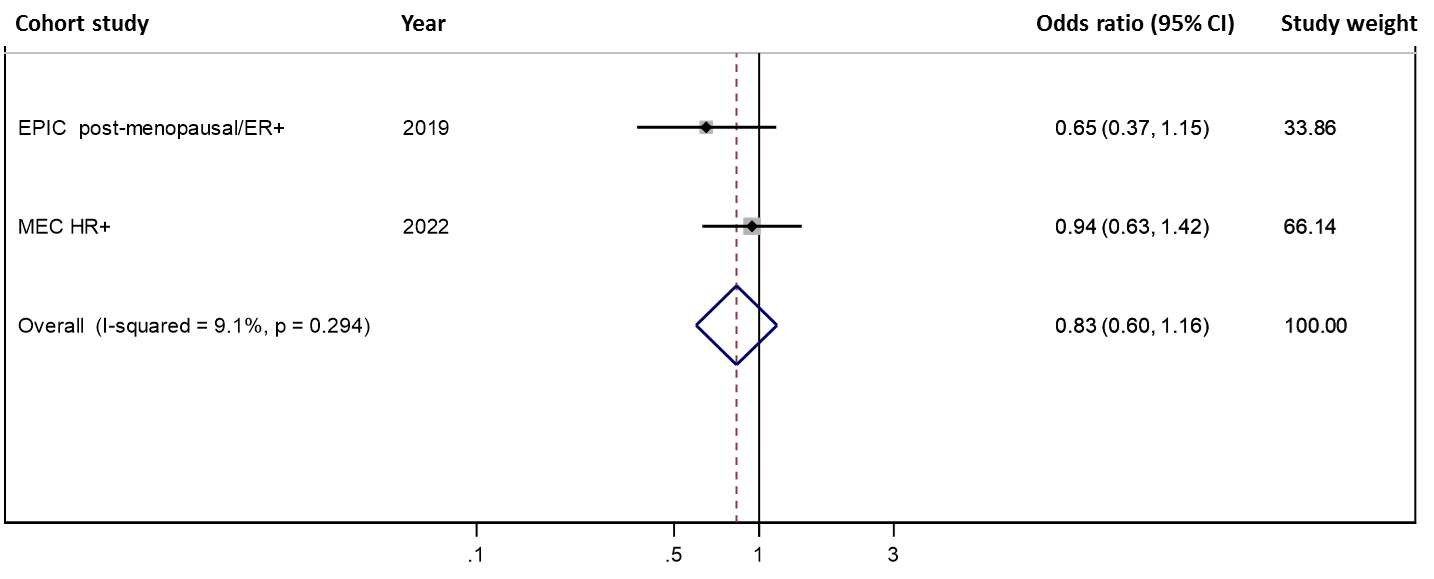

Supplement: Supplementary file 1 — Additional file 1. Supplemental methods, tables, and figures. [file 13058_2023_1693_MOESM1_ESM.docx]
